# Calcium/Calmodulin Dependent Protein Kinase II Bound to NMDA Receptor 2B Subunit Exhibits Increased ATP Affinity and Attenuated Dephosphorylation

**DOI:** 10.1371/journal.pone.0016495

**Published:** 2011-03-15

**Authors:** John Cheriyan, Parimal Kumar, Madhavan Mayadevi, Avadhesha Surolia, Ramakrishnapillai V. Omkumar

**Affiliations:** 1 Molecular Neurobiology Division, Rajiv Gandhi Centre for Biotechnology, Thiruvananthapuram, Kerala, India; 2 Molecular Biophysics Unit, Indian Institute of Science, Bangalore, Karnataka, India; 3 National Institute of Immunology, New Delhi, India; Universidade Federal do Rio de Janeiro, Brazil

## Abstract

Calcium/calmodulin dependent protein kinase II (CaMKII) is implicated to play a key role in learning and memory. NR2B subunit of N-methyl-D-aspartate receptor (NMDAR) is a high affinity binding partner of CaMKII at the postsynaptic membrane. NR2B binds to the T-site of CaMKII and modulates its catalysis. By direct measurement using isothermal titration calorimetry (ITC), we show that NR2B binding causes about 11 fold increase in the affinity of CaMKII for ATPγS, an analogue of ATP. ITC data is also consistent with an ordered binding mechanism for CaMKII with ATP binding the catalytic site first followed by peptide substrate. We also show that dephosphorylation of phospho-Thr^286^-α-CaMKII is attenuated when NR2B is bound to CaMKII. This favors the persistence of Thr^286^ autophosphorylated state of CaMKII in a CaMKII/phosphatase conjugate system *in vitro*. Overall our data indicate that the NR2B- bound state of CaMKII attains unique biochemical properties which could help in the efficient functioning of the proposed molecular switch supporting synaptic memory.

## Introduction

Calcium/calmodulin dependent protein kinase II (CaMKII) is a protein found enriched in the brain. Owing to its unique autoregulatory ability, CaMKII is implicated to play a major role in the molecular mechanisms underlying learning and memory. In the postsynaptic compartment, Ca^2+^ influx through N-methyl-D-aspartate receptor (NMDAR) activates CaMKII, following which, it translocates from cytosol to postsynaptic density (PSD) and binds to NMDAR subunit 2B (NR2B) [1]–[4]. This interaction has been shown to be important for the induction of long term potentiation (LTP) which is a cellular correlate for learning and memory [5]. The disruption of this interaction has been shown recently to produce deficits in hippocampal LTP and spatial learning [6]. Binding of CaMKII to NR2B, by a non-catalytic site called T-site, enables it to remain autonomously active [7]. In addition, the interaction between CaMKII and NR2B through the T-site has been found to modulate the kinetics of catalysis by the enzyme [8]. It was proposed that CaMKII in combination with protein phosphatase 1 (PP1), a phosphatase enriched in PSD, can form a Ca^2+^-sensitive molecular switch that can respond with specificity to the type of Ca^2+^ signals and provide stability to molecular memories [9]–[12]. Since, binding of CaMKII to NR2B is essential for LTP, we hypothesized that NR2B-bound CaMKII might contribute to this switch [5], [6]. Therefore we have studied the biochemical properties of NR2B-bound CaMKII *in vitro* towards attaining a better understanding of the regulatory mechanisms affecting the CaMKII-phosphatase switch.

In the present study, by a direct measurement of binding affinity using isothermal titration calorimetry (ITC), we show that the affinity of the ATP analogue, ATPγS, for CaMKII increases significantly in the presence of NR2B as shown by the change in value of the association constant, K_a_. From a separate set of experiments, we also present data to reveal how NR2B favours the persistence of Thr^286^ autophosphorylated form of CaMKII. The implications of these findings for the efficient functioning of the CaMKII-PP1 switch are discussed.

## Results

### ATP saturation kinetics of NR2B bound CaMKII

**Figure 1:**
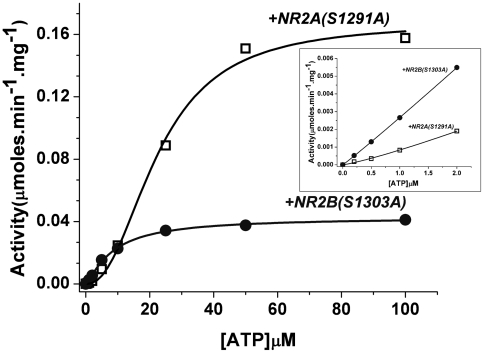
ATP saturation of NR2B bound CaMKII. CaMKII was preincubated with either GST-NR2B (S1303A) (•) or GST-NR2A (S1291A) (□) and the activity was assayed using phosphorylatable GST-NR2A (WT) as substrate. The data were fitted to the Hill equation and plotted using Origin software. The inset shows initial concentration points plotted separately to highlight the enhancement in activity of CaMKII in the presence of NR2B. Data represents three similar experiments.

We have previously reported that CaMKII shows enhanced activity at low [ATP] in the presence of saturating concentrations of non-phosphorylatable GST-NR2B (S1303A) [8]. When pretreated with subsaturating concentrations of GST-NR2B (S1303A) also, CaMKII showed enhanced activity at lower [ATP] compared with control CaMKII pretreated with non-phosphorylatable GST-NR2A (S1291A) (Fig. 1 inset). It has previously been shown by GST pull down assay that NR2A sequence does not bind to the T-site of CaMKII [3], [ 8]. The activity of the enzyme achieves saturation at very low [ATP] in presence of NR2B sequence and stays constant for a broad range of [ATP] whereas in the absence of NR2B the activity attained saturation only at much higher [ATP]. This indicates an enhancement in affinity for ATP in the presence of NR2B sequence. Interestingly, the maximal activity observed in the presence of NR2B sequence was much lower than that in its absence (Fig. 1).

### Order of binding of substrates to CaMKII

**Figure 2:**
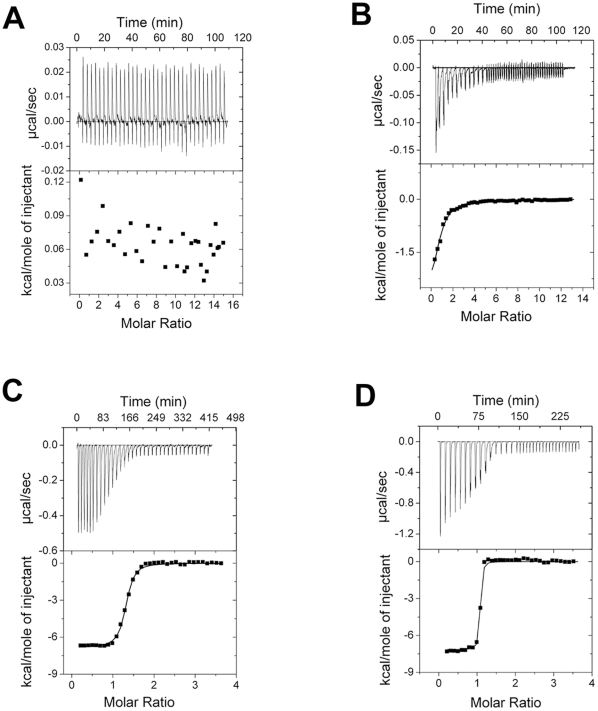
ITC profiles of ATPγS titrations on α-CaMKII. Blank titrations with ATPγS in the absence of any protein (A), titrations with ATPγS on calmodulin activated CaMKII (B), titrations as in B in the presence of GST-NR2A (C) and titrations as in B in the presence of GST-NR2B (D) are shown. Molar ratio is that of ligand (ATPγS) to macromolecule (α-CaMKII) after injection.

We resorted to ITC measurements to study binding of substrates to CaMKII. For this purpose ATPγS was titrated against calmodulin activated CaMKII in the absence of any CaMKII binding partner as well as in their presence (Fig. 2). We found that although the signals were weak and irregular in the absence of any protein ligand, a clear pattern indicating binding could be seen (Fig. 2B). The signals seemed to suggest that the binding of ATPγS to CaMKII accompanies slow conformational rearrangements in CaMKII. The values of the titration parameters obtained are as follows: N = 0.72±0.067, K_a_ = 8.29×10^4^±1.06×10^4^ M^−1^, ΔH = −3428±379.6 cal/mol, ΔS = 10.8 cal/mol*K (Fig. 2B). A subsequent titration with protein or peptide substrate detected heat changes due to specific binding corresponding to ternary complex formation (data not shown). Consistent with these results, titration by ATPγS in the presence of protein ligands also showed strong signals of heat change (Fig. 2C, 2D). When the order of titrating substrate was reversed, by titrating protein substrate first, non-specific signals were obtained indicating the lack of any detectable binding (data not shown). This tends to suggest that the substrate binding on CaMKII follows an ordered mechanism in which ATP binds first followed by the protein substrate. Considering that there are conflicting reports regarding the order of binding of substrates to CaMKII [13]–[15], our experiments provide direct binding data which is in agreement with the earlier reports that have indicated that CaMKII follows an ordered ternary complex formation mechanism [14], [15].

### Larger association constant for ATPγS binding to CaMKII in the presence of NR2B

The ATP analogue, ATPγS was used in order to prevent phosphorylation of the proteins in the titration experiments. ATPγS titrations on CaMKII in the presence of protein substrates yielded strong signals for the heat change which decreased and approached the baseline (Fig. 2C, 2D). This suggests the formation of a stable enzyme-substrate ternary complex proportional in amount to the titrated substrate. Since CaMKII follows an ordered ternary complex mechanism for its catalysis as shown in the previous section, wherein ATP comes first in the order of binding, the ATPγS titration data obtained here can be considered as characteristic of ATP binding to CaMKII (Fig. 2C, 2D) [14], [15]. The binding reactions were exothermic in the temperature range of the experiments. The thermodynamic parameters and the K_a_ values obtained from the titrations in the presence of GST-NR2A and GST-NR2B are shown in Table 1. The K_a_ value for ATPγS binding to CaMKII in presence of GST-NR2B is about 11 fold higher than that in the presence of GST-NR2A. The difference in the K_a_ values in all probability arises from the increase in the affinity of CaMKII for ATPγS, induced by NR2B as a result of its binding to CaMKII at the T site. These findings are consistent with data obtained from activity measurements (Fig. 1).

**Table 1:** ITC data for ATPγS binding to CaMKII.

Protein substrate	N	K_a_ (M^−1^)	ΔH (cal/mol)	ΔS (cal/mol*K)	ΔG (cal/mol)
*GST-NR2A*	1.28±0.0065	2.22×10^6^±2.7×10^5^	−6814±59.15	5.8	−8499
*GST-NR2B*	1.04±0.0021	2.51×10^7^±4.3×10^6^	−7439±32.36	8.48	−9909

Titrations were done by adding ATPγS to CaMKII that has already been equilibrated with either GST-NR2A or GST-NR2B. Chi square values for the titrations in the presence of GST-NR2A and GST-NR2B were 2.25×10^4^ and 8679 respectively.

We carried out titrations at two different temperatures. At 30°C there was a tendency for precipitation during the titration while the titrations at 20°C were found to be ideal for the measurements. Table 1 shows the summary of the thermodynamic parameters obtained at 20°C. As can be seen from Table 1, the ΔH, ΔS and ΔG values are greater in magnitude for ATPγS binding to CaMKII in the presence of NR2B compared to ATPγS binding to CaMKII in presence of NR2A.

### Dephosphorylation of phospho-Thr^286^-CaMKII is resisted in the presence of NR2B

**Figure 3:**
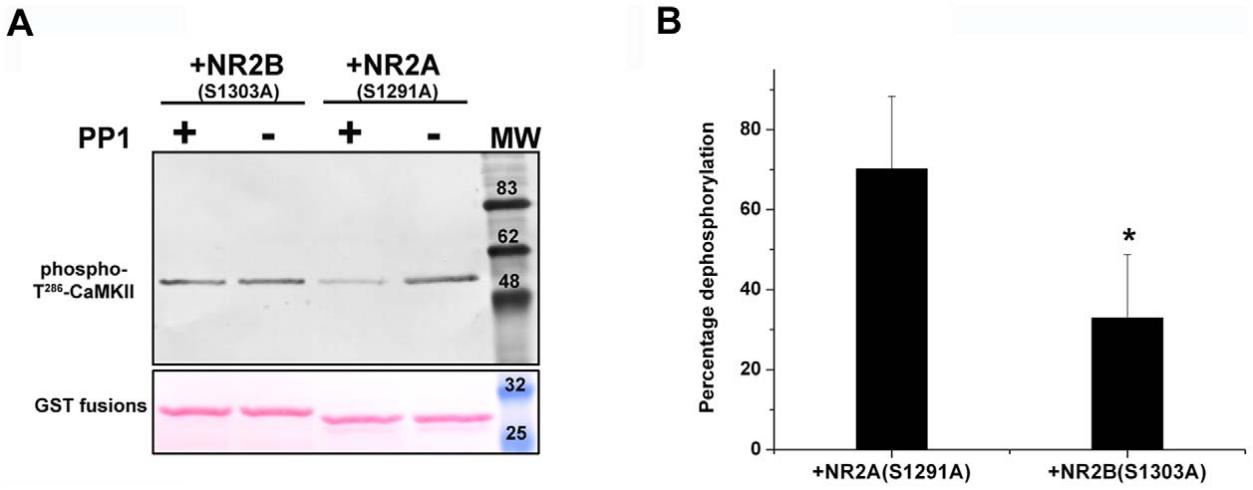
Enhancement in the level of phospho-Thr^286^ of α-CaMKII in the presence of GST-NR2B (S1303A) in the CaMKII/phosphatase coupled system. CaMKII and PP1 were maintained simultaneously active in the reaction for 5 min and the sample was then analysed by western blotting. Panel A shows the Western blot probed using anti-phospho-Thr^286^-α-CaMKII antibody and GST fusion protein bands stained by Ponceau S. Values obtained by densitometry from four experiments were used for calculating percentage dephosphorylation shown as bar graphs in panel B. In each set, the band intensity of the sample that was not treated with PP1 [PP1 (−)] shown in A was taken as 100%. (*p value<0.05).

**Figure 4:**
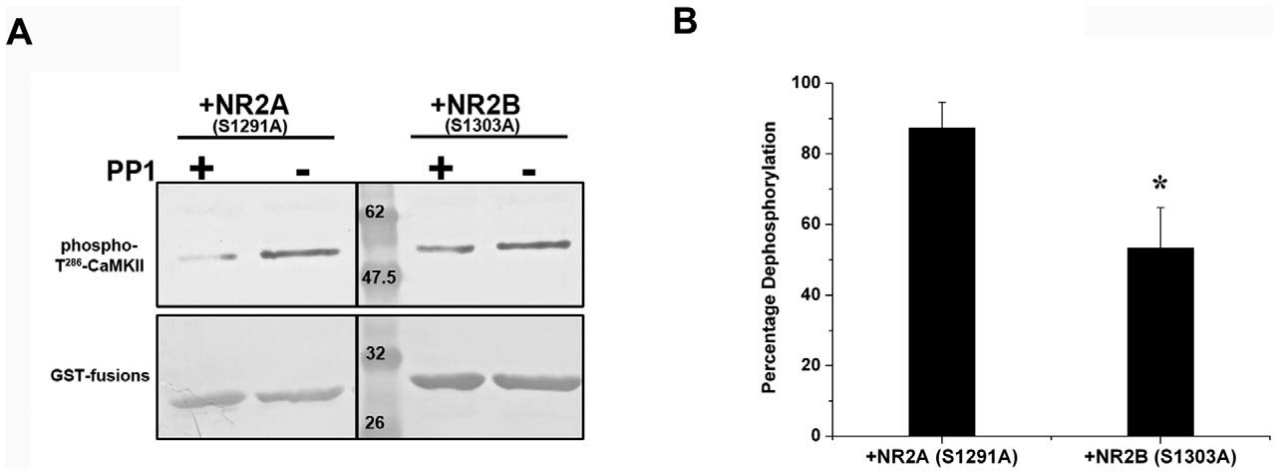
Reduced susceptibility of phospho-Thr^286^ of α-CaMKII to dephosphorylation in the presence of GST-NR2B (S1303A). CaMKII was initially autophosphorylated in the absence of phosphatase as described in methods. Subsequently dephosphorylation by PP1 was carried out after stopping the kinase reaction with staurosporine. Representative Western blot is shown in panel A. Quantified values from four determinations (two experiments) of phospho-Thr^286^ levels on Western blots were used for calculating percentage dephosphorylation shown as bar graphs in panel B. In each set, the band intensity of the sample that was not treated with PP1 [PP1 (−)] shown in A was taken as 100%. (*p value<0.005).

We reconstituted a system *in vitro* to mimic the CaMKII-phosphatase switch that has previously been proposed [10]–[12]. A coupled reaction of autophosphorylation alongside dephosphorylation of CaMKII-Thr^286^ was performed considering the physiological possibility of simultaneous autophosphorylation and dephosphorylation. The reactions were carried out either in the presence of GST-NR2B (S1303A) or GST-NR2A (S1291A). It was very interesting to find that the presence of NR2B sequence caused an increase in the level of autophosphorylated CaMKII when compared to that in the presence of the homologous NR2A sequence (Fig. 3 and Fig. S2). Similar results were obtained even when the duration of the reaction was varied (data not shown). Since the final autophosphorylation level observed at the termination of the reaction would be the result of the forward and reverse processes, it is possible that the increase in phospho-Thr^286^ might be due to the enhanced rate of autophosphorylation in the presence of NR2B or due to reduced dephosphorylation owing to the inability of the phosphatase to access phospho-Thr^286^ when NR2B resides at the T-site or both. We tried to resolve this by another set of experiments in which the autophosphorylation and dephosphorylation reactions were decoupled by stopping the kinase reaction with staurosporine before phosphatase treatment. We found that even if the autophosphorylation reaction is stopped, the amount of phospho-Thr^286^ was high in those samples with GST-NR2B (S1303A) indicating that the presence of NR2B segment inhibited dephosphorylation of phospho-Thr^286^-CaMKII by PP1(Fig. 4).

## Discussion

CaMKII present in PSD is believed to form the CaMKII-phosphatase switch that is involved in supporting synaptic memory mechanisms such as LTP [10]–[12]. This involves the system of CaMKII and phospho-CaMKII that are continuously interconverted by the kinase activity of CaMKII and the phosphatase activity of PP1. The binding of CaMKII to NR2B present in the PSD, during the induction of LTP [5], [6], might recruit it to the CaMKII-PP1 switch. We speculated that, NR2B-bound CaMKII might acquire properties that very well may modulate this switch. Hence we investigated the effects of NR2B on both the kinase reaction as well as on the dephosphorylation of phospho-Thr^286^.

We show that NR2B enhances the phosphorylation activity of α-CaMKII at low ATP concentrations (Fig. 1). Earlier kinetics studies had suggested a decrease in the K_m_ value of ATP when GST-NR2B was the peptide substrate [8]. The CaMKII autophosphorylation kinetics also showed a similar effect on ATP binding in the presence of non-phosphorylatable GST-NR2B (S1303A) [8]. In an enzyme reaction mechanism, the apparent K_m_ value is representative of the several steps, each expectedly having different rate constants [16]. It is a constant derived from the Michaelis-Menten equation and is calculated indirectly from the measured rate of the enzymatic reaction. It could be altered by influences at any one of the different stages of the process. One of the reasons for the reduction in K_m_ could be an increase in the affinity of the enzyme for ATP. Direct evidence of the increase in the enzyme's affinity for ATP in the presence of GST-NR2B can be obtained by measuring the association constant, K_a_ for ATP. Hence, ITC which is one of the most direct ways to measure binding parameters was used [17].

In order to measure the parameters of nucleotide binding prior to the phosphate transfer step, ATPγS, an ATP analogue resistant to hydrolysis was used to prevent the reaction from proceeding to completion which otherwise would result in a large heat change that will mask the heat change due to binding. Moreover, the interference of Thr^286^-autophosphorylation reaction can also be prevented by this approach. Throughout the titration experiments we had used calmodulin activated CaMKII so that the binding events subsequent to calmodulin binding alone are measured. This also avoids any reciprocal modulation of binding between ATP and calmodulin [18], [19]. Analysis by ITC revealed that the binding of ATPγS to CaMKII is favored by almost 11 fold increase in affinity due to the presence of NR2B as seen by increase in K_a_ value (Table 1). Such insights into the functional regulation of CaMKII become possible by ITC analysis of CaMKII holoenzyme [19], [20].

The K_m_ values obtained for ATP by the enzyme kinetics experiments were in the micromolar range. The K_a_ values obtained for ATPγS binding also gives K_d_ (K_d_ = 1/K_a_) [21] in the micromolar range although the values are still lower than the reported K_m_ values [8], [22]. The difference between the values obtained in the presence of either NR2A or NR2B using ITC are similar to the observations made in biochemical studies with NR2B inducing a higher affinity for the nucleotide binding (Fig. 1) [8]. The extent of difference in K_a_ values obtained in the present study is however higher (∼11 fold) compared to the differences in K_m_ values for ATP obtained by enzyme kinetics (∼6 fold) [8]. This difference might have arisen since in the microcalorimetry experiments, the non-hydrolysable analogue, ATPγS was used. In addition, the molar ratios of GST-NR2B (S1303A) to CaMKII in the ITC experiments were different from that in the kinetics experiments reported earlier [8]. However, the modulatory action of NR2B does exist at different molar ratios of GST-NR2B (S1303A) to CaMKII (Fig. 1) [8].

The binding order of substrates to CaMKII has been investigated in the past. Data from enzyme kinetics experiments favoring an ordered mechanism as well as a random mechanism have been reported warranting further studies on the order of substrate binding to CaMKII [13]–[15], [23]. Our measurements of binding using microcalorimetry detect enthalpy changes upon titration of ATPγS to CaMKII (Fig. 2B) but not upon titration of the NR2A or NR2B fusion proteins to CaMKII (data not shown). This supports an ordered substrate binding mechanism in which the catalytic cycle of CaMKII involves the formation of the enzyme-ATP binary complex followed by the enzyme-ATP-protein substrate ternary complex [14], [15]. However, we do not exclude the possibility of entropically driven binding with undetectable enthalpy changes. Since the heat changes detected are dependent on the concentration of ATPγS added, the difference in binding parameters obtained between the titrations in presence of NR2A and NR2B should also be due to differences in the ATP-binding step (Fig. 2C, 2D, Table 1). The pattern of ATP concentration dependence of the phosphorylation activity was consistent with enhanced affinity in the presence of NR2B sequence (Fig. 1). It is interesting to note that the ternary complex formation involving GST-NR2B has a larger −ΔG value compared to the complex formation with GST-NR2A. The increase in ATPγS binding to CaMKII, in the presence of NR2B, is driven by the electrostatic interactions between ATPγS and CaMKII (a high ΔH value for ATPγS binding to NR2B bound CaMKII). At the same time, an increased entropy change (ΔS) suggests a structural rearrangement facilitating ATPγS binding. The enhancement of ATP binding indicates that the modulation by NR2B favors catalysis in a positive way. We note that the K_a_ value obtained for titration by ATPγS in the absence of any peptide is smaller by more than an order of magnitude compared to that in the presence of peptide substrate (Figs. 2B, 2C, 2D). This might be a consequence of the formation of the ternary complex in presence of the peptide substrate.

The change in catalytic parameters of CaMKII upon NR2B binding may serve its role in supporting synaptic memories. The complex of CaMKII with NR2B may be considered as a new enzyme form that is sensitive to lower ATP concentrations and is also stable, owing to the persistent nature of NR2B binding to T-site [24]. Although it is generally believed that intracellular [ATP] is in the millimolar range and hence is not limiting, ATP concentrations at synapses are subject to significant variations because of the high rates of ATP dependent processes. High likelihood of an ATP gradient formation at spines is also reported [25]. Moreover, it has also been reported that the ATP required in the PSD, when necessary, can be synthesized by the glycolytic machinery resident in the PSD which is subject to modulation by several metabolites and hence, can be variable [26]. Variations in [ATP] can, in principle, lead to fluctuations in the kinase reaction in the switch if CaMKII is in the free form. The constant rate of reaction exhibited by the NR2B-bound CaMKII over a wide range of ATP concentrations can thereby provide stability to the switch against variations in ATP concentrations (Fig. 1). As it may not be feasible to have all the CaMKII subunits bound by NR2B, it could be hypothesized that the NR2B bound subunits in a CaMKII holoenzyme act as the initiators of autophosphorylation reaction at low ATP concentrations.

To address the effect of NR2B on the CaMKII-phosphatase system, we used an assay system in which CaMKII and PP1 were both active and the resulting level of phospho-Thr^286^–CaMKII was measured. This led to an interesting observation that in such a system, the level of Thr^286^ autophosphorylation remains high when NR2B is present (Fig. 3). This could be due to the higher autophosphorylation rate in the presence of NR2B as reported earlier [8]. In addition, it is also possible that there is a reduction in the rate of dephosphorylation of CaMKII in presence of NR2B. In order to test whether the dephosphorylation reaction is affected, staurosporine was added to stop the kinase activity before the addition of phosphatase. This led to the finding that the dephosphorylation reaction was significantly reduced in the presence of NR2B, thus suggesting that there is an additional regulatory mechanism other than enhancement of kinase activity by NR2B. Any direct effect of the GST-fusion proteins on PP1 was ruled out by pNPP hydrolysis assay of the activity of PP1 in presence of the fusion proteins (Fig. S3). It may be speculated that the binding of NR2B to the T-site could be causing hindrance for free access of phosphatases towards phospho-Thr^286^ of CaMKII. The conformation of the Thr^286^ containing motif may undergo significant changes upon binding of NR2B similar to what was reported earlier for Ca^2+^/calmodulin binding [27]. The resultant resistance of NR2B bound CaMKII subunits to dephosphorylation could very well be assumed to be one of the reasons behind the reported structural mechanism that prevents CaMKII dephosphorylation in PSD [28].

**Figure 5:**
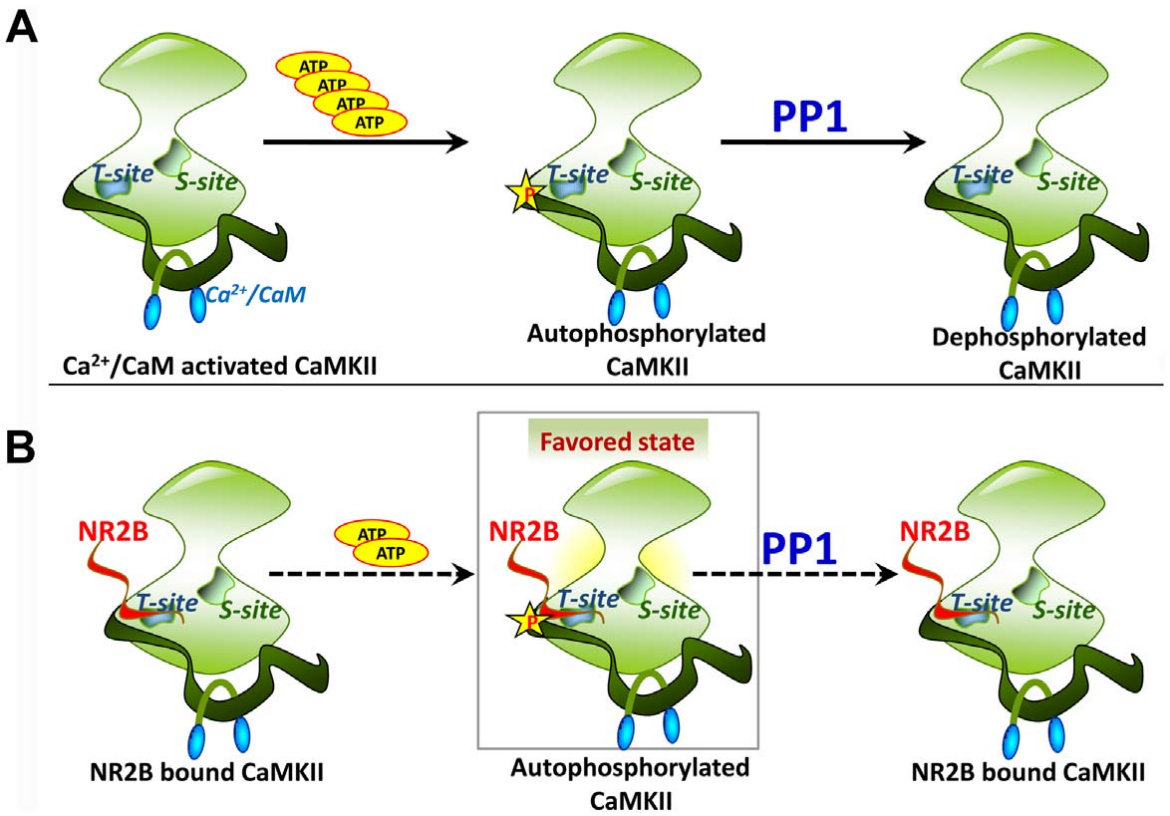
Schematic diagram showing the coupled autophosphorylation-dephosphorylation reaction *in vitro* that represents the CaMKII-PP1 switch. A) The normal course of Thr^286^ autophosphorylation of CaMKII and its dephosphorylation by PP1. B) Binding of NR2B to the T-site of CaMKII increases the ATP binding affinity. NR2B binding also makes the enzyme less susceptible to dephosphorylation by PP1. The dotted arrows represent slower reaction rates. Both the autophosphorylation and dephosphorylation reaction rates are reduced for the NR2B-bound CaMKII. This helps to maintain the Thr^286^-autophosphorylated state in a CaMKII-PP1 switch with minimal consumption of ATP.

One of the characteristics proposed for the CaMKII-phosphatase switch is its energy efficient operation. Based on kinetic considerations, the rates of both the autophosphorylation and dephosphorylation reactions have been assumed to be low for the proper functioning of the switch. Since the dynamic maintenance of the switch consumes ATP, low reaction rates for the forward and reverse reactions help in minimizing consumption of ATP and thus energy efficient functioning of the switch [10]. Our data shows that the binding of NR2B causes reduction in the rates of the phosphorylation (Fig. 1, 25 µM to 100 µM) and dephosphorylation reactions (Fig. 4), thereby providing a biochemical mechanism that permits the functioning of the kinase-phosphatase switch in an energy efficient manner (Fig. 5). In summary, our study reports the direct biochemical effects of NR2B binding to CaMKII that might confer stability and energy efficiency to the CaMKII-phosphatase switch in PSD. The data presented can contribute to building of quantitative biochemical models of CaMKII function at synapses.

## Materials and Methods

### Materials

Amicon Ultra centrifugal devices were from Millipore. PIPES, Hepes, IPL-41 insect cell culture medium, antibiotic/antimycotic cocktail, fetal bovine serum (FBS), protease inhibitor cocktail, β-mercaptoethanol, calmodulin purified from bovine testes, etc. were from Sigma-Aldrich, USA. ATPγS was from Roche or Sigma. PD-10 desalting columns, calmodulin-Sepharose, glutathione-Sepharose, etc. were from Amersham/GE Healthcare, USA. Reduced glutathione was from Sisco Research Laboratories, India or Calbiochem, USA. *Sf*21 cells were from National Centre for Cell Science, Pune, India. For our experiments, we have used GST fusions of peptide sequences based on the phosphorylation sites of NMDAR subunits NR2A and NR2B and termed them as GST-NR2A and GST-NR2B respectively [8], [29]. Corresponding non-phosphorylatable mutants [GST-NR2B (S1303A) and GST-NR2A (S1291A)] were also used. All the GST fusion proteins were expressed in *E. coli*.

### Expression and purification of α-CaMKII

Expression of α-CaMKII in insect cells was carried out as described before [30]. Adherent cultures of *Sf*21 cells in 175 cm^2^ flasks were infected with the stock of recombinant baculovirus encoding WT-α-CaMKII. The infected cells were harvested 72 hours post infection. Purification of the expressed protein was carried out as explained earlier. Each batch of purification had insect cell pellets from 15 flasks. The cell pellets were resuspended in lysis buffer containing 50 mM PIPES, pH 7.0, 5% betaine, 1 mM EGTA, 1 mM EDTA and 1× complete protease inhibitor cocktail (Sigma).

In the first step of purification the lysate was loaded onto a 75 ml bed volume phosphocellulose cation exchanger column pre-equilibrated with equilibration buffer (50 mM PIPES, pH 7.0, 100 mM NaCl, 1 mM EGTA and 1× protease inhibitor cocktail). The bound protein was eluted with elution buffer (50 mM PIPES, pH 7.0, 500 mM NaCl, 1 mM EGTA and 1× protease inhibitor cocktail). The eluate having CaMKII activity was used for affinity purification on CaM-Sepharose column as described before [8].

A 20 ml bed volume CaM-sepharose column was used for affinity purification. Equilibration buffer contained 40 mM Hepes, pH 7.3, 0.1 M NaCl, 10% glycerol and 2 mM CaCl_2_. The flow-through was collected and was reloaded once. A high salt wash with equilibration buffer containing 1 M NaCl was given followed by wash with equilibration buffer before elution in the buffer having 40 mM Hepes pH 7.3, 0.5 M NaCl, 5% glycerol, and 3.5 mM EGTA.

### Expression and purification of GST fusion proteins

The GST fusion proteins were expressed in BL21 DE3 strain of *E.coli* as described before [8]. The expressed proteins were purified by affinity chromatography using glutathione-Sepharose column. The crude lysate containing the expressed protein was loaded onto the column pre-equilibrated with PBS. The bound protein was eluted in buffer containing 40 mM Hepes pH 7.3, 0.5 M NaCl, 5% glycerol, 3.5 mM EGTA and 10–20 mM reduced glutathione.

### Concentrating the purified proteins and buffer exchanges

We adopted a simplified procedure in which the ionic constituents (4 mM CaCl_2_ and 15 mM MgCl_2_) and 0.5 mM β-mercaptoethanol required in the final titration experiments were added to the purified CaMKII before concentrating the protein using Amicon Ultra centrifugal devices with a molecular weight cut-off of 100 kDa. The dodecameric α-CaMKII with a molecular mass of approximately 600 kDa, will be retained by the filter during concentration. The filtrate obtained by this method will have all the constituents except the enzyme and can thereafter be used to reconstitute the ligand solution.

The filtrate collected during concentration of the enzyme was used to equilibrate the purified GST fusion proteins which had been eluted in the same buffer with added glutathione. The buffer exchange of purified GST fusion proteins was carried out using PD10 gel filtration columns after concentrating the GST fusion protein to a reduced volume in 10 kDa cut-off Amicon Ultra centrifugal devices.

CaMKII subunit concentration achieved was about 48–50 µM while GST fusion proteins were concentrated to 416–460 µM (Fig. S1).

### Protein concentration

Concentrations of the purified proteins were estimated by the bicinchoninic acid (BCA) method.

### ATP saturation kinetics with NR2B treated CaMKII

Kinetic analyses were carried out as described earlier [8]. CaMKII was preincubated along with Ca^2+^/CaM (2 mM/27 U per µl), and 3.2 µM of either GST-NR2B (S1303A) or GST-NR2A (S1291A). This was used as the enzyme source for the assay. Assay had a final concentration of 2 mM CaCl_2_ and 2.7 U/µl CaM. [γ-^32^P] ATP at concentrations ranging from 0.2 µM–100 µM was used to carry out the assay. The phosphorylated bands were detected from the autoradiogram and were quantified by densitometry using QuantityOne software.

### Isothermal titration calorimetry

Isothermal titration calorimetry (ITC) experiments were done using a VP-ITC system (Microcal Inc.). All the experiments presented were conducted at 20°C while trials at 30°C were also done. The buffer used was 40 mM Hepes, pH 7.3, 0.5 M NaCl, 5% glycerol, 4 mM CaCl_2_, at least 4 U/µl calmodulin, 0.5 mM β-mercaptoethanol, 3.5 mM EGTA and 15 mM MgCl_2_ except for the blank titration (Fig. 2A) in which calmodulin was absent. ATPγS was reconstituted in the same buffer. Multiple titrations were carried out at various concentrations to optimize the conditions. ATPγS taken in the syringe had a concentration of 1 to 1.5 mM. Injection parameters for the ligand were 6–8 µl/injection with time spacing of 200–700 seconds depending on the progress of the titrations. The final protein concentrations used for the experiments were 21 µM subunit concentration of α-CaMKII and 82 µM GST-NR2A or GST-NR2B. Three sets of ATPγS titrations were performed; 1) ATPγS titration on CaMKII, 2) ATPγS titration on CaMKII with GST-NR2A, and 3) ATPγS titration on CaMKII with GST-NR2B. Titrations were initiated after incubating CaMKII in the buffer to ensure binding of calmodulin. The titration experiments involving the GST fusion proteins were carried out in two steps, with the GST-fusion protein substrate being titrated first followed by the ATPγS titration.

### Analysis of Calorimetric data

Data obtained from the titrations was analyzed using Origin^Tm^ 7.0 software. Before analysis of the data, the heat changes accompanying ATPγS binding to CaMKII alone were subtracted from the individual data of enthalpy change accompanying ATPγS binding to NR2B or NR2A saturated CaMKII. The data were fit to single binding site model. From the curve, values for stoichiometry of binding (N), association constant (K_a_) and enthalpy of binding (ΔH) were obtained. Change in entropy (ΔS) was obtained using the equation :(ΔG_b_ = ΔH_b_−TΔS), where ΔG_b_ = −RTlnK_a_; R and T represent the gas constant and the absolute temperature (in Kelvin), respectively.

### Dephosphorylation of phospho-Thr^286^-CaMKII

#### a) Simultaneous autophosphorylation and dephosphorylation of CaMKII

The reactions were carried out in the presence of non-phosphorylatable GST-NR2A or GST-NR2B. A preincubation step having 0.8 µM CaMKII, 6.4 µM of either GST-NR2B (S1303A) or GST-NR2A (S1291A), 2 mM CaCl_2_ and 27 U/µl CaM was carried out before the assay. The reaction mix for assay was similar to that of the autophosphorylation reaction described earlier except for the addition of phosphatase, PP1 [8]. Each assay tube contained final concentrations of 50 mM Tris (pH 8.0), 10 mM MgCl_2_, 0.4 mM EGTA, 1.3 mM CaCl_2_, 6.7 U/µl CaM, 0.2 mg/ml BSA, 1 mM MnCl_2_, 0.9 µM of ATP, 0.2 µM CaMKII and either 1.6 µM GST-(S1291A)-NR2A or 1.6 µM GST-(S1303A)-NR2B in the presence or absence of 3.75 units of PP1 in a total volume of 20 µl. The reaction duration was mostly 5 minutes, but 1 minute, and 15 minutes durations were also tried. The reactions were started by the addition of ATP and were stopped by the addition of 5× SDS sample buffer. The reaction samples were resolved in a 10% SDS-PAGE and western blotting was carried out to monitor Thr^286^ autophosphorylation. A mouse monoclonal anti-phospho-Thr^286^-α-CaMKII primary antibody was used in conjunction with alkaline phosphatase conjugated secondary antibody. Experiments using γ-^32^P-ATP were also performed. The reaction samples were resolved in a 10% SDS-PAGE gel which was later dried and exposed to phosphor screen and was subsequently scanned in a BioRad PhosphorImager.

#### b) Dephosphorylation after autophosphorylation

Autophosphorylation reaction was carried out as mentioned above for 30 seconds without including phosphatase or the GST fusion proteins and the reaction was terminated by adding 10 µM staurosporine, a kinase inhibitor. After stopping the autophosphorylation reaction, aliquots of the autophosphorylated CaMKII were incubated with GST-NR2B (S1303A) or GST-NR2A (S1291A) separately, in the same buffer. The dephosphorylation reaction in these samples was initiated by the addition of PP1 and the reaction was allowed for 30 minutes. The reactions were stopped by quick freezing by transferring to −80°C followed by addition of 5× SDS sample buffer.

## Supporting Information

Figure S1 A) Representative SDS-PAGE showing purified α-CaMKII (1 µg). B) SDS-PAGE of purified and concentrated GST-NR2A and GST-NR2B used for ITC experiments. Molecular sizes are indicated in kDa. 16 µg of purified GST-NR2A and 15 µg of purified GST-NR2B were loaded.
(TIF)

Figure S2 Enhancement in the level of phospho-Thr^286^ of α-CaMKII in the presence of GST-NR2B (S1303A) in the CaMKII/phosphatase coupled system. Autoradiogram of autophosphorylated CaMKII (^32^P-labeled) is shown. The duration of the reaction was 1 minute. Reactions were started by addition of 0.7 µM [γ-^32^P] ATP as described in methods. Data represents at least three similar experiments.
(TIF)

Figure S3 PP1 activity assay using pNPP (para-Nitrophenyl Phosphate) hydrolysis to investigate the effect of GST fusion proteins on the activity of PP1. A 50 µl reaction was set up which had 1× PP1 buffer (50 mM HEPES, pH 7.0, 0.1 mM EDTA, 5 mM DTT and 0.025% Tween-20), 1 mM MnCl_2_, 50 µM pNPP, 0.34 µM GST-(S1291A)-NR2A or 0.27 µM GST-(S1303A)-NR2B and 2.5 U of PP1. The experiment was carried out in a 96 well plate. The reaction mixture was incubated for 10 minutes at 30°C. The reaction was stopped by the addition 0.5 M EDTA and the absorbance was measured at 405 nm wavelength in an automated microplate reader. The activity was unaffected in the presence of GST fusion proteins but was significantly reduced by the phosphatase inhibitor, okadaic acid.
(TIF)
