# Supplementary figures and images for: Calcium/Calmodulin Dependent Protein Kinase II Bound to NMDA Receptor 2B Subunit Exhibits Increased ATP Affinity and Attenuated Dephosphorylation
Source: PLoS One. 2011 Mar 15;6(3):e16495. doi: 10.1371/journal.pone.0016495 (PMC3057968; doi:10.1371/journal.pone.0016495)

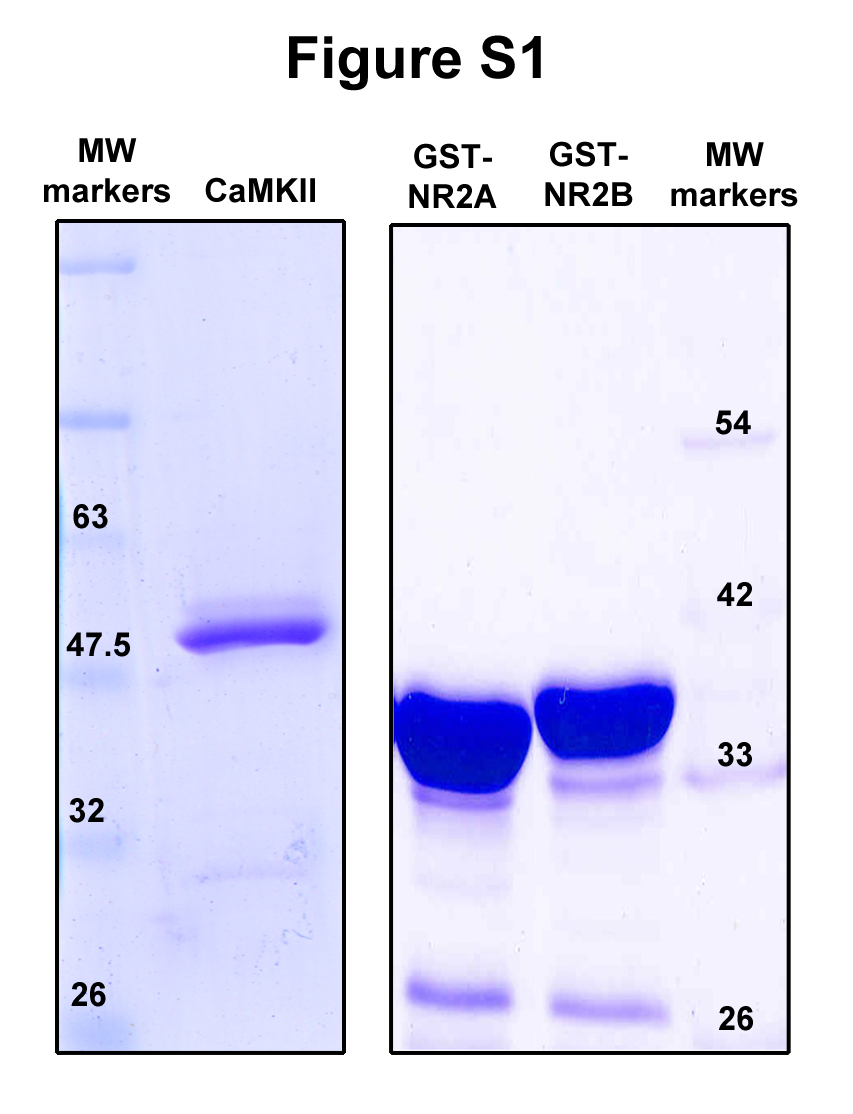

Supplement: Figure S1 — A) Representative SDS-PAGE showing purified α-CaMKII (1 µg). B) SDS-PAGE of purified and concentrated GST-NR2A and GST-NR2B used for ITC experiments. Molecular sizes are indicated in kDa. 16 µg of purified GST-NR2A and 15 µg of purified GST-NR2B were loaded. (TIF) [file pone.0016495.s001.tif]

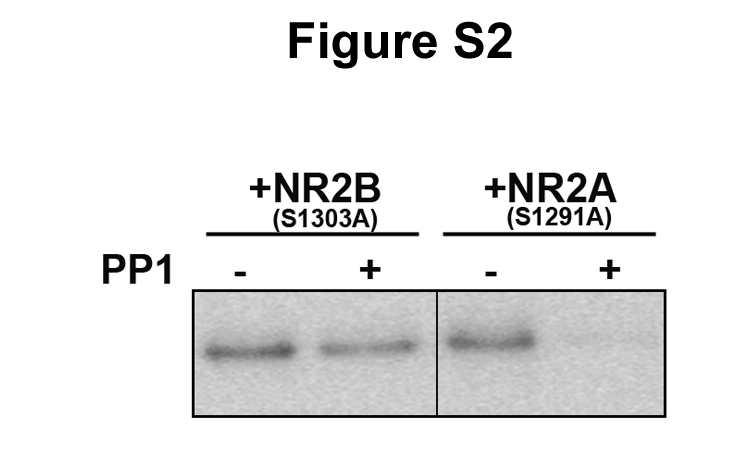

Supplement: Figure S2 — Enhancement in the level of phospho-Thr286 of α-CaMKII in the presence of GST-NR2B (S1303A) in the CaMKII/phosphatase coupled system. Autoradiogram of autophosphorylated CaMKII (32P-labeled) is shown. The duration of the reaction was 1 minute. Reactions were started by addition of 0.7 µM [γ-32P] ATP as described in methods. Data represents at least three similar experiments. (TIF) [file pone.0016495.s002.tif]

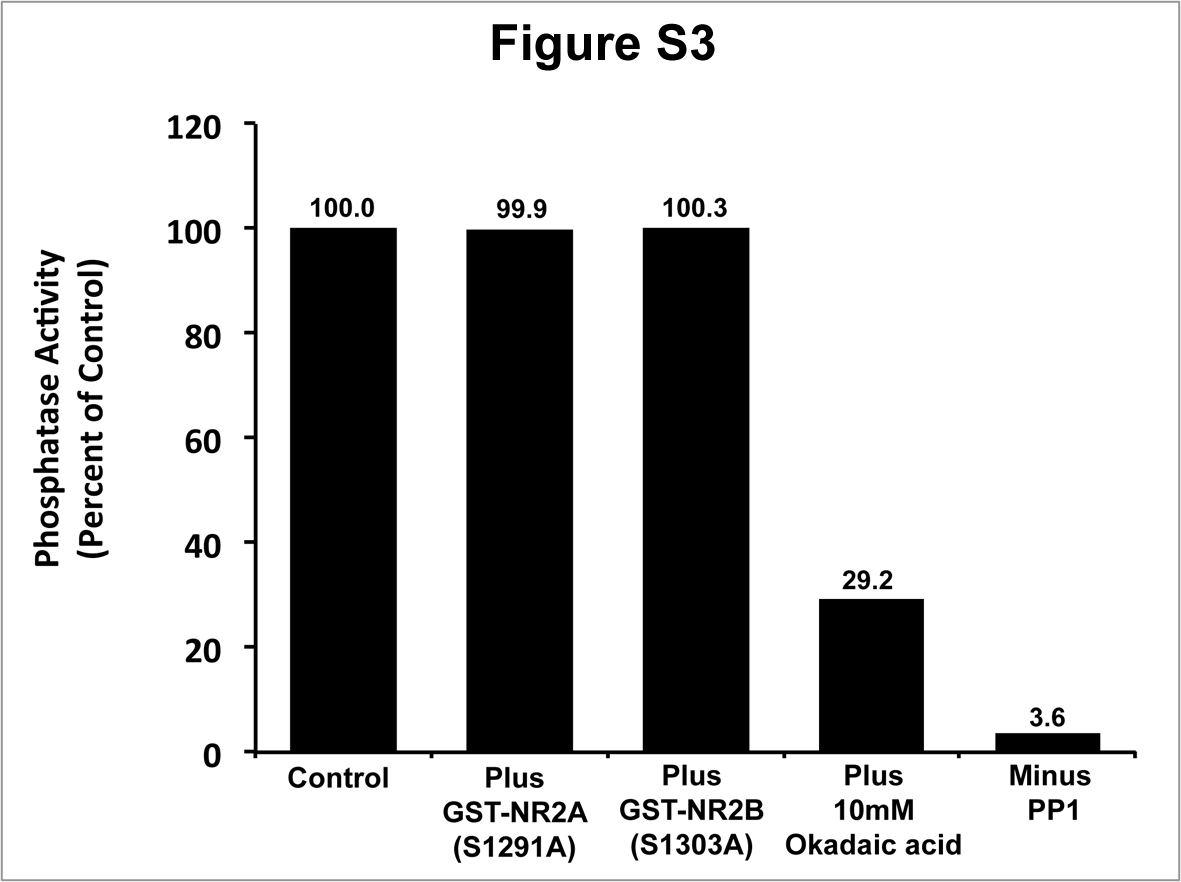

Supplement: Figure S3 — PP1 activity assay using pNPP (para-Nitrophenyl Phosphate) hydrolysis to investigate the effect of GST fusion proteins on the activity of PP1. A 50 µl reaction was set up which had 1× PP1 buffer (50 mM HEPES, pH 7.0, 0.1 mM EDTA, 5 mM DTT and 0.025% Tween-20), 1 mM MnCl2, 50 µM pNPP, 0.34 µM GST-(S1291A)-NR2A or 0.27 µM GST-(S1303A)-NR2B and 2.5 U of PP1. The experiment was carried out in a 96 well plate. The reaction mixture was incubated for 10 minutes at 30°C. The reaction was stopped by the addition 0.5 M EDTA and the absorbance was measured at 405 nm wavelength in an automated microplate reader. The activity was unaffected in the presence of GST fusion proteins but was significantly reduced by the phosphatase inhibitor, okadaic acid. (TIF) [file pone.0016495.s003.tif]
